# Allocation of emergency medical resources for epidemic diseases considering the heterogeneity of epidemic areas

**DOI:** 10.3389/fpubh.2023.992197

**Published:** 2023-02-24

**Authors:** Bin Hu, Guanhua Jiang, Xinyi Yao, Wei Chen, Tingyu Yue, Qitong Zhao, Zongliang Wen

**Affiliations:** ^1^School of Public Health, Xuzhou Medical University, Xuzhou, China; ^2^School of Management, Xuzhou Medical University, Xuzhou, China; ^3^Department of Logistics and Supply Chain Management School of Business, Singapore University of Social Science, Singapore, Singapore; ^4^Affiliated Hospital of Xuzhou Medical University, Xuzhou, China

**Keywords:** epidemic diseases, time-varying demand forecasting model, emergency medical resource distribution decision model, resource allocation, ε-constraint method, weighting sum method

## Abstract

**Background:**

The resources available to fight an epidemic are typically limited, and the time and effort required to control it grow as the start date of the containment effort are delayed. When the population is afflicted in various regions, scheduling a fair and acceptable distribution of limited available resources stored in multiple emergency resource centers to each epidemic area has become a serious problem that requires immediate resolution.

**Methods:**

This study presents an emergency medical logistics model for rapid response to public health emergencies. The proposed methodology consists of two recursive mechanisms: (1) time-varying forecasting of medical resources and (2) emergency medical resource allocation. Considering the epidemic's features and the heterogeneity of existing medical treatment capabilities in different epidemic areas, we provide the modified susceptible-exposed-infected-recovered (SEIR) model to predict the early stage emergency medical resource demand for epidemics. Then we define emergency indicators for each epidemic area based on this. By maximizing the weighted demand satisfaction rate and minimizing the total vehicle travel distance, we develop a bi-objective optimization model to determine the optimal medical resource allocation plan.

**Results:**

Decision-makers should assign appropriate values to parameters at various stages of the emergency process based on the actual situation, to ensure that the results obtained are feasible and effective. It is necessary to set up an appropriate number of supply points in the epidemic emergency medical logistics supply to effectively reduce rescue costs and improve the level of emergency services.

**Conclusions:**

Overall, this work provides managerial insights to improve decisions made on medical distribution as per demand forecasting for quick response to public health emergencies.

## 1. Introduction

Since the turn of the twenty-first century, there have been numerous outbreaks of large-scale infectious diseases throughout the world. An epidemic occurs when an infectious disease spreads quickly and affects a significant number of individuals. Epidemic infections can spread widely in a short period, usually within 2 weeks or less (1). The most dangerous epidemics include SARS (2003), H1N1 influenza (2009), Middle East Respiratory Syndrome (2012), Ebola virus in West Africa (2014), Zika virus in Brazil (2015), and the continuing COVID-19, which broke out in late 2019 and rapidly became a pandemic, affecting the lives of millions at a global scale. With the emergence of the Novel Coronavirus variant, the number of new coronavirus cases in the world remains high every day. According to the latest real-time statistics from the WHO, as of 17:34 am Central European Time on December 1 (0:34 am Beijing time on December 2), the cumulative number of confirmed COVID-19 cases in the world has reached 63,957,2,819, with 6,615,258 cumulative deaths. These outbreaks pose a major threat to human physical and mental health, as well as to world economic progress.

Emergency medical logistics in response to public health emergencies is very important, but the research in this area is still inadequate (2). This work reviews the related literature by first focusing on prediction of the number of infected person infectious diseases and then discussing the approaches to logistics distribution for an emergency.

The use of the mathematical model to analyze the kinetic behavior of diseases dates back to Daniel Bernoulli's (3) 1760 research on the inoculation of smallpox infectious disease, which is known as the Bernoulli equation. Enko et al. (4) developed the discrete infectious disease model based on the binomial distribution for the first time by collecting the data on scarlet fever and measles and published the chain binomial model of scarlet fever and measles in 1889. The twentieth century saw the emergence of deterministic research on infectious illness models. Hamer (5) developed the dynamic measles model of herd immunity, which is a discrete state model with a bilinear infection rate. For the first time, a mass effect was proposed in the model, and it was assumed that the effective infection rate of individuals was proportional to the number of susceptible individuals. Ross (6) developed a differential equation model for the first time through the study of malaria transmission rules, demonstrating that malaria transmission could be controlled when the number of mosquitoes was reduced to a certain threshold and defined the standard infection rate and basic regeneration number. Kermack et al. (7) established a susceptible -infected-recovered (SIR) model after jointly studying the transmission trend of the Black Death in London, England, in 1665 and the plague in Mumbai, India, in 1906. Subsequently, the susceptible-infected-susceptible model was established in 1932 (8). Samsuzzoha et al. (9) established a susceptible-vaccinated-exposed-infected-recovered (SVEIRS) model, a diffuse zonal epidemic model of vaccination based on the SEIRS model, to investigate the impact of vaccination and transmission dynamics of influenza epidemics. Chinazzi et al. (10) predicted the influence of travel restrictions on the domestic and international transmission of an epidemic disease using a global congregational disease transmission model, and they find the quarantine in Wuhan slowed the overall progress of the epidemic in mainland China by 3–5 days and had a more significant impact on the international scale. Jumpen et al. (11–13) established a susceptible-exposed-infected-quarantined-recovered (SEIQR) model to simulate the evolution of the epidemic from the perspective of patient isolation, and used sensitivity analysis to investigate the impact of parameter uncertainty on the prediction of disease transmission. Yang et al. (14) updated the SEIR model to account for population flow and used deep learning methods to properly forecast the spread of an outbreak in China. Jia et al. (15) projected the relative frequency and geographical distribution of new coronavirus type 2 infection in mainland China before February 19, 2020, using the Wuhan population distribution. The publication of these scientific study findings established a highly effective theoretical and practical foundation for the efficient execution of epidemic prevention and control. Primarily, most scholars studied the laws of the epidemic disease transmission from two perspectives: the evolution of the outbreak population and the effect of related parameters on the development of the outbreak. They used the related mathematical theory and methods to prove the stability of the epidemic spread model, basic reproductive number, and threshold value for the existence of equilibrium of the model itself. These mathematical models can be used to describe the dynamic changes associated with various diseases and to forecast outbreak demand. In this paper, the system dynamics model is used to study the law of epidemic disease transmission.

For the dispatching and distribution of emergency supplies problem, scholars have established optimization models for allocating emergency materials in the event of a public health emergency, such as an outbreak of infectious diseases (16–26). Some of these scholars overlooked the impact of the evolution of infectious disease epidemics on supplies (16–21). Miniguano-Trujillo et al. (27) developed a multi-periodic integer programming optimization model and heuristic algorithm for allocating appropriate time to therapists for various patient types. A mixed-integer linear programming (MILP) model was established to achieve equity. Numerical results showed that treatment resources allocation based on population ratio is suboptimal. Implementing such a resource allocation policy may decrease the total number of infections and deaths, resulting in high costs that must be paid for equity. Repoussis et al. (28, 29) developed the optimal allocation of emergency medical resources in mass casualty events. Because it is critical to prioritize the allocation of emergency medical resources, the author turned the triage problem with limited resources into an ambulance route problem model to determine patients' evacuation sequence and destination hospital. To address the problem and find the optimal solution, an algorithm based on the column generation method was proposed. Pu (30) proposed a MILP model to tackle the allocation of patients to hospitals and treatment sequencing difficulties in mass casualty incidents, intending to effectively allocate limited resources in the response phase. Simultaneously, the model was built to minimize the total response time and flow time needed to treat all patients, and it was solved using precise and MILP-based heuristics. Ray (31) developed a model for emergency relief material transportation under various constraints to optimize transportation expenses. In general, current research results indicate that, within the context of public health emergency studies, emergency logistics management is a significant research topic, and its study has profound theoretical and practical implications, demonstrating the necessity and importance of emergency logistics management. Additionally, it contributes significantly to the reduction of fatalities and property damage caused by emergencies. However, most studies (32, 33) focus on the one-time distribution of supplies and dispatch of emergency supplies, and rarely consider the emergency in epidemic areas, dynamic distribution of supplies, or actual situations in which different local medical treatment capabilities exist against the background of an epidemic and resulting in varying influences on the epidemic transmission evolution trend. After the outbreak of infectious diseases, the epidemic degree varies by epidemic location, and the demand for supplies in the epidemic area is determined by the number of confirmed infections.

The primary objective of this study is to quantify the actual situation in the epidemic area to distribute supplies, and help decision-maker to make best choices on the allocation of limited emergency medical resources to the appropriate places and quantities to halt the outbreak and mitigate its effects.

To be specific, this paper is based on human data collected in each of the affected areas. A particular point in time in the historical data is selected as the basis point. The personnel situation in each epidemic area at the decision time is forecasted based on the information at the basis point using the modified infectious disease model. The number of infected and hospitalized groups is simulated in each area to forecast patients' demand for emergency medical resources. Under the assumption that the demand for emergency medical resources for patients with infectious diseases and inpatients is known, we define emergency indicators for each epidemic area and develop a bi-objective optimization model to determine the optimal medical service allocation plan by maximizing the weighted demand satisfaction rate and minimizing the total vehicle travel distance.

In summary, this paper takes sudden infectious public events as the background, emphasizes emergency logistics schedule optimization, and presents an emergency rescue logistics model based on infectious diseases transmission mechanism. First, based on the transmission rules of infectious diseases combined with the local medical treatment capability, the modified susceptible-exposed-infected-recovered (SEIR) model are proposed to predict the material demand of each epidemic area during the entire emergency rescue stage. Second, a mixed-integer programming model for multi-stage and multi-cycle emergency rescue logistics scheduling in multi-epidemic areas is built with fairness (34) and timeliness in mind, and the route selection of rescue vehicles and allocation of medical resources are optimized. Finally, using the linear weighting and ε-constraint method, the MILP model of multi-objective emergency rescue logistics scheduling is transformed into a single-objective model, and LINGO software and YALMIP toolbox MATLAB program are used to validate the numerical examples.

## 2. Methods

### 2.1. Model assumptions

1. The local population is relatively steady, regardless of migration, natural birth rate, or death rate.
2. The infected individual requires pharmacological therapy following hospitalization.
3. Individuals who have recovered get permanent immunity.
4. It is assumed that the functional relationship between the demand for emergency medical resources and the number of infected individuals in the epidemic area is known.
5. It is assumed that the demand for supplies in the epidemic area and reserves of relief centers can be forecasted, as well as the number of and geographic location of epidemic areas.
6. This paper does not consider the number of vehicles, transportation mode, transportation capacity limits, or other related factors while implementing quarantine policies during an epidemic.

### 2.2. The time-varying demand forecasting model

According to the unique characteristics of the spread of infectious diseases, the precise allocation of emergency supplies during an epidemic can be summarized as follows: (1) During the early stages of an epidemic, it is necessary to quantify the extent of the emergency in epidemic areas. The epidemic locations are diverse, and each area has a unique crisis circumstance. (2) Because supplies demand are highly correlated with the number of confirmed infections, it is vital to forecast the demand in the epidemic area based on the number of infected people.

**Figure 1:**
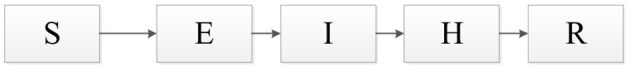
Schematic diagram of state transition.

Based on the characteristics of the initial spread of infectious disease, hence the SEIHR warehouse models are created. We categorize the affected area's population into six subgroups: susceptible (S), exposed (E), infective (I) who have developed symptoms following the incubation period but have not been hospitalized or isolated, hospitalized infected (H), and recovered (R). N = S + E + I + H + R is used to calculate the total population in each epidemic area. Figure 1 shows the transfer of this model between these epidemic classes. Table 1 shows the definitions of the time-varying forecasting of the medical resources model.

**Table 1:** Time-varying forecasting of medical resources mathematical notations.

**Notation**	**Definition**
S	Familiar and vulnerable susceptible people
*E*	Exposed people
*I*	Infectious people
*H*	Hospitalized infected people
*R*	Recovered people
*J*	A set of epidemic areas, *j*=1,2,3,..*n*;
*S*_*j*_(*t*)	Numbers of common and vulnerable susceptible people in area *j* at the moment *t*
*E*_*j*_(*t*)	Numbers of common and vulnerable exposed people in area *j* at the moment *t*
*I*_*j*_(*t*)	Numbers of common and vulnerable infectious people in area *j* at the moment *t*
*H*_*j*_(*t*)	Numbers of common and vulnerable hospitalized infected people in area *j* at the moment *t*
*R*_*j*_(*t*)	Numbers of common and vulnerable recovered people in area *j* at the moment *t*
$\beta_{j}^{1}$	Exposure rate of susceptible people *S* in contact with infectious people *I* in area *j*
$\beta_{j}^{2}$	Exposure rate of susceptible people *S* in contact with exposed people *E* in area *j*
$\beta_{j}^{3}$	Exposure rate of susceptible people *S* in contact with asymptomatic infected people *E* in area *j*
$\sigma_{j}^{-1}$	Mean duration of latency (days)in area *j*
δ_*j*_	The local medical treatment capacity mainly determines the rate at which infected people in area*s j* change to hospitalized patients
γ_*j*_	Common and vulnerable recovery rate in area *j*

The susceptible population (S) reduce as a result of exposure to exposed, infected, and asymptomatic infected people, and they will enter the incubation period of exposure (moving to class E). As shown in formula 1.

The exposed population (E) become infected (moving to class I) with evident symptoms. As shown in formula 2.

The infected population (I) enter the inpatient stage (moving to class H) according to the local medical treatment capability. As shown in formula 3.

After treatment, the hospitalized population (H) reduces and enter the recovery population (moving to class R). As shown in formula 4.

The recovery population is immune to this disease.

### 2.3. SEIHR model


(1) $$\begin{array}{l} \frac{dS_{j}}{dt}=-\beta_{j}^{1}S_{j}\left(t\right)I_{j}\left(t\right)-\beta_{j}^{2}S_{j}\left(t\right)E_{j}\left(t\right)\; \end{array}$$

(2) $$\begin{array}{l} \frac{dE_{j}}{dt}=\beta_{j}^{1}S_{j}\left(t\right)I_{j}\left(t\right)+\beta_{j}^{2}S_{j}\left(t\right)E_{j}\left(t\right)\;-\sigma_{j}E_{j}\left(t\right) \end{array}$$

(3) $$\begin{array}{l} \frac{dI_{j}}{dt}=\sigma_{j}E_{j}\left(t\right)-\delta_{j}I_{j}\left(t\right) \end{array}$$

(4) $$\begin{array}{l} \frac{dH_{j}}{dt}=\delta_{j}I_{j}\left(t\right)-\gamma_{j}H_{j}\left(t\right) \end{array}$$

(5) $$\begin{array}{l} \frac{dR_{j}}{dt}=\gamma_{j}H_{j}\left(t\right) \end{array}$$

### 2.4. Emergency medical resource distribution decision model

Mixed-integer linear programming (MILP) is used to develop a deterministic resource allocation model. Table 2 shows the definitions of the Emergency medical resource distribution decision model.

**Table 2:** Emergency medical resource distribution decision mathematical notations.

I	A set of supply points, *i*=1,2,3,.. *m*;
T	A set of time, one rescue cycle per day
$X_{j}^{t}$	The demand satisfaction rate of emergency medical resources in epidemic area *j* at the moment *t*
*r*_*ij*_	Vehicle travel distance from supply point *i* to epidemic area *j* at the moment *t*
$P_{j}^{t}$	The stock of emergency medical resources that epidemic area j before moment t begins
$X_{j,min}^{t}$	The lower threshold of requirement satisfaction rate of emergency medical resources in epidemic area *j* at moment *t*,0$<X_{j,min}^{t}<$1
$Q_{i}^{t}$	The stock of emergency medical resources at the supply point before moment t begins
$\omega_{j}^{t}$	The degree of urgency in the demand for emergency medical resources in epidemic areas
*K*	The upper limit of the number of supply points for transporting medical resources to epidemic areas

#### 2.4.1. Notation


(6) $$\omega_{j}^{t}=\frac{I_{j}(t)\text{+}H_{j}(t)}{\sum_{j\in J}\left(I_{j}(t)\text{+}H_{j}(t)\right)}$$

#### 2.4.2. Decision variables

Based on the descriptions and assumptions of the abovementioned models, the following multi-objective emergency relief supply distribution decision model can be established for each time cycle, to maximize the weighted sum of the demand satisfaction rates and minimize travel distance:

$X_{ij}^{t}$: The quantity of emergency medical resources allocated from supply point *i* to epidemic area *j* at the moment *t*

$y_{ij}^{t}$: 0–1 variable: if emergency medical resources are delivered from supply point *i* to epidemic area *j* at the moment *t*, it is 1; otherwise, it is 0.

#### 2.4.3. Objective function

Objective 1: To maximize the weighted sum of demand satisfaction rates for each period.


(7) $$\max Z_{1}=\sum_{j\in J}\omega_{j}^{t}X_{j}^{t}$$

Objective 2: To minimize the sum of driving distances per period.


(8) $$\min Z_{2}=\sum_{i\in I}\sum_{j\in J}y_{ij}^{t}r_{ij}$$

#### 2.4.4. Constraints

Subject to


(9) $$X_{j}^{t}=\min\left\{\frac{P_{j}^{t}+\sum_{i\in I}X_{ij}^{t}}{D_{j}^{t}},1\right\}\forall j\in J,t\in T$$

(10) $$\begin{array}{l} X_{j,\min}^{t}\leq X_{j}^{t},\forall j\in J,t\in T \end{array}$$

(11) $$\begin{array}{l} \underset{j\in J}{\sum }X_{ij}^{t}\leq Q_{i}^{t},\forall i\in I,t\in T \end{array}$$

(12) $$\begin{array}{l} X_{ij}^{t}\left(1-y_{ij}^{t}\right)=0,\forall i\in I,j\in J,t\in T \end{array}$$

(13) $$\sum_{j\in J}X_{ij}^{t}\leq \max\left\{D_{j}^{t}-P_{j}^{t},0\right\},\forall j\in J,t\in T$$

(14) $$\begin{array}{l} \underset{i\in I}{\sum }y_{ij}^{t}\leq K,\;\forall i\in I,t\in T \end{array}$$

(15) $$\begin{array}{l} y_{ij}^{t}\in \left\{0,1\right\},\forall i\in I,j\in J,t\in T \end{array}$$

(16) $$\begin{array}{l} X_{ij}^{t}\geq 0,\forall i\in I,j\in J,t\in T \end{array}$$

Constraint (9) is the formula for calculating the demand satisfaction rate. When the supply exceeds the demand, the demand satisfaction rate should be equal to one.

Constraint (10) establishes a minimal demand satisfaction rate for all epidemic areas, which reflects the fairness principle.

According to constraint (11), the quantity of emergency medical resources delivered from the central distribution center shall not exceed its stock.

Constraint (12) indicates the relationship between these two variables, that is, only when the quantity of goods $X_{ij}^{t}$ dispatched from the supply point to the epidemic area is not equal to 0, then the $y_{ij}^{t}$ is equal to 1.

The purpose of constraint (13) is to prevent the supply point from receiving more materials than required.

Constraint (14) demonstrates that one epidemic area can only be served by **K** supply point to prevent wasting of transport capacity.

Constraints (15) and (16) are constraints on variable values.

### 2.5. Model solution

The key to solving multi-objective (35–38) problems is to convert the multi-objective function into a single-objective programming problem using the weighted method, constraint method, and mixed-method, which is solved using the traditional single-objective programming method. The ε-constraint method is an exact approach that is capable of generating non-extreme efficient solutions. An ε-constraint method also performs well in mixed-integer multi-objective problems.

In this paper, the multi-objective problem is standardized using min-max first, and then the maximization objective function is converted into the minimization objective.


(17) $$\min Z_{1}^{\prime }=-Z_{1}=-\sum_{j\hat{I}J}\omega_{j}^{t}X_{j}^{t}$$

#### 2.5.1. ε-Constraint method

An ε-constraint method is a well-known approach used for solving multi-objective problems. This method is to convert a multi-objective problem into a single-objective problem, where only one objective is optimized and the remaining objectives are treated as constraints. The general form of this algorithm is defined by Eq. (18), where X denotes the feasible set of the mathematical model:


(18) $$\begin{array}{l} \min f_{1}x\\ subject\;to\;x\in X \end{array}$$

$$\begin{array}{l} f_{2}x\leq \varepsilon_{2}\\ .....\\ f_{n}x\leq \varepsilon_{n} \end{array}$$

#### 2.5.2. Weighting sum method

Weighted sum method is the most frequently used technique to evaluate efficient solutions for a deterministic multi-objective optimization problem. The multi-objective problem is converted into a single-objective problem using the linear weighted grouping method, and assigning a weight to each sub-objective based on its importance. The overall objective function is:


(19) $$\min Z=\lambda_{1}\left(\frac{-\sum_{j\in J}\omega_{j}^{t}X_{j}^{t}-Z_{1,\min}^{\prime }}{Z_{1,\max}^{\prime }-Z_{1,\min}^{\prime }}\right)+\lambda_{2}\left(\frac{\sum_{i\in I}\sum_{j\in J}y_{ij}^{t}r_{ij}-Z_{2,\min}}{Z_{2,\max}-Z_{2,\min}}\right)$$

According to Formula (16), when the supply of emergency medical resources is sufficient to fulfill demand, the first term on the right end has a constant value of 1. In this case, the model is simplified as the optimization model to minimize the total driving distance. When the supply of medical resources for all points does not exceed the demand, the optimization model is reformulated into a MILP problem.

Constraint (9) is transformed into:


(20) $$\begin{array}{l} X_{j}^{t}=\frac{P_{j}^{t}+\underset{i\in I}{\sum }X_{ij}^{t}}{D_{j}^{t}}\leq 1,\forall j\in J,t\in T \end{array}$$

### 2.6. Numerical experiments

#### 2.6.1. Base case

Suppose that eight epidemic areas (numbered 1, 2, 3, 4, 5, 6, 7, 8) require immediate assistance as a result of the emergence of the epidemic. Four supply points (numbered A, B, C, and D) can provide emergency medical resources for the epidemic areas, with storage of A = 2,400, B = 2,500, C = 1000, and D = 1100, respectively. One supply point can only supply one epidemic area. Because the propagation of the epidemic differs from other situations and has an infectious time, it is difficult to determine the exact start date of the crisis. As a result, a date in the past is chosen as the start time. Different epidemic areas' start time information is shown in Table 3. Distances (in units/km) between the eight epidemic areas and the four supply points are shown in Table 4.

**Table 3:** Coordinates and number of residents at resident demand node I.

**Number*J***	**1**	**2**	**3**	**4**	**5**	**6**	**7**	**8**
*S*_0_	8million	5million	3million	4million	2million	3.5million	2.5million	5.5million
*E*_0_	5,000	4,000	2,000	3,000	1,000	3,000	2,000	5,000
*I*_0_	600	500	300	400	200	400	300	600
*H*_0_	180	125	120	100	20	60	60	125
*R*_0_	156	95	126	75	22	23	36	110
$\beta_{j}^{1}$	0.02	0.02	0.02	0.02	0.02	0.02	0.02	0.02
$\beta_{j}^{2}$	0.01	0.01	0.01	0.01	0.01	0.01	0.01	0.01
$\beta_{j}^{3}$	0.2	0.2	0.2	0.2	0.2	0.2	0.2	0.2
σ_*j*_	0.2	0.2	0.2	0.2	0.2	0.2	0.2	0.2
δ_*j*_	0.1	0.05	0.2	0.05	0.1	0.05	0.04	0.1
γ_*j*_	0.2	0.15	0.3	0.15	0.1	0.1	0.1	0.15
α_*j*_	0.02	0.02	0.02	0.02	0.02	0.02	0.02	0.02
$P_{j}^{2}$	193	105	89	108	92	85	57	172

**Table 4:** Vehicle travel distance from the supply point to the epidemic area.

***r*_*ij*_**	**1**	**2**	**3**	**4**	**5**	**6**	**7**	**8**
A	390	370	880	750	145	200	235	515
B	620	635	1,100	1,050	355	240	205	540
C	550	440	500	305	625	700	800	340
D	450	400	780	725	240	175	325	220

#### 2.6.2. Experiments on the base case

The sensitivity analysis of the calculation results is conducted below, and the effect of adjusting the minimum demand satisfaction rates in the model is studied. When we change the number of supply points in an epidemic area, the results are also different.

## 3. Results

### 3.1. Base case

**Figure 2:**
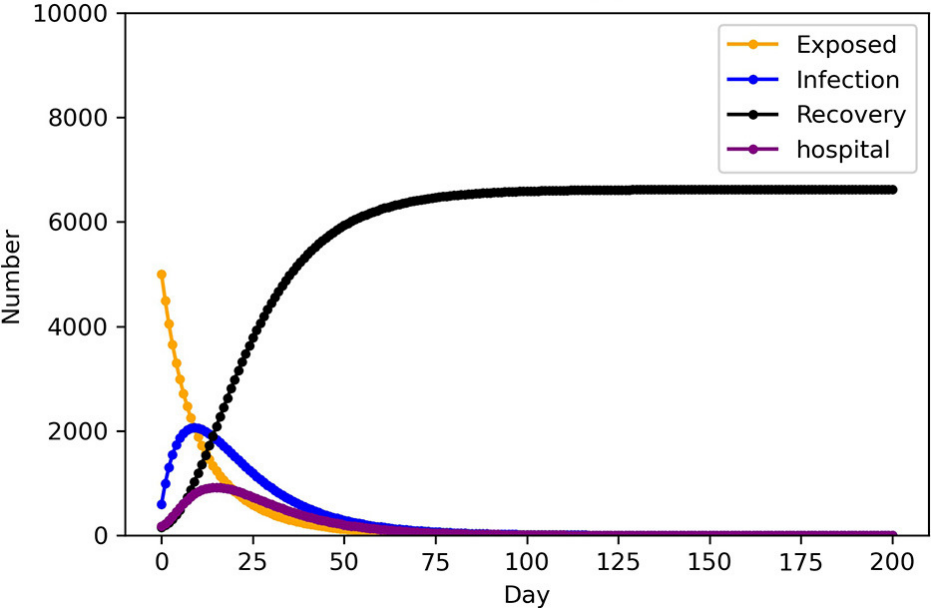
Epidemic trend in scenario 1 (Asymptomatic not included) of epidemic area 1.

**Figure 3:**
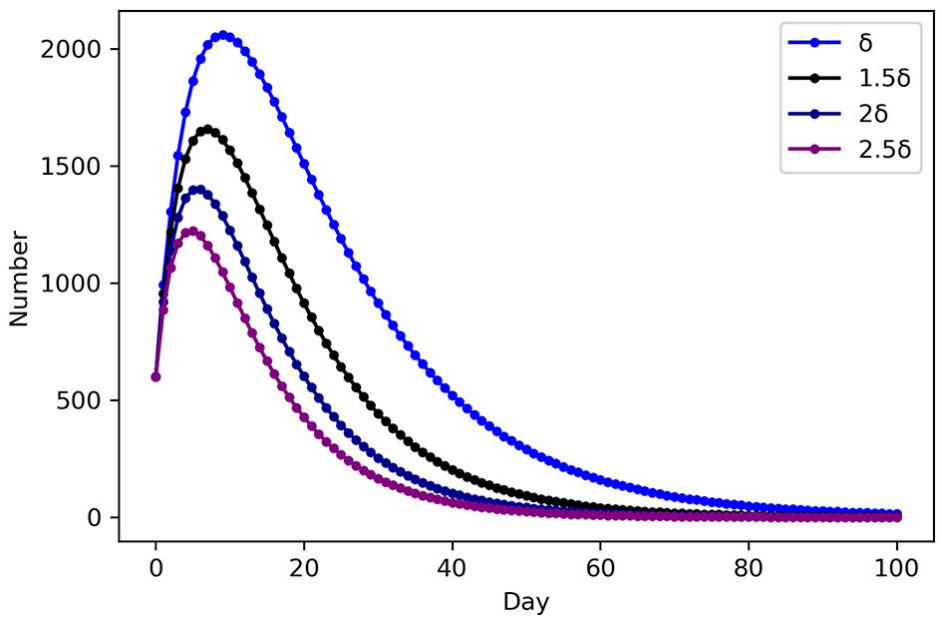
Epidemic trend in scenario 2 (Asymptomatic included) of epidemic area 1.

As indicated in Table 5, the SEIHR model is used to calculate the number of infected people, level of emergency, and total medical demand in each epidemic area after 2 days, Figure 2 show the variation in population numbers over time in epidemic area 1. Medical and health intervention is manifested by the level of medical treatment when infectious diseases occur, whether the medical resources are sufficient and complete, and whether all the patients can be collected when the number of infected patients increases rapidly, so that the patients can get timely and effective treatment. To improve the ability to treat patients can be regarded as the rate at which infected people in area*s j* change to hospitalized patients of infectious disease model. δ = (δ, 1.5δ, 2δ, 2.5δ), to analyze the impact on the number of infected people. Control variable method was adopted, remaining parameters remained unchanged, and SEIHR model was used to simulate the development of infected persons in Region 1, as shown in Figure 3.

**Table 5:** Current information of the epidemic area.

**Epidemic area**	**1**	**2**	**3**	**4**	**5**	**6**	**7**	**8**
*I*_0_	1,307	1,139	497	877	327	877	624	1,307
*H*_0_	286	166	192	131	65	108	83	265
$\omega_{j}^{2}$	0.19307	0.15816	0.08351	0.12217	0.04751	0.11938	0.08569	0.19052
$D_{j}^{2}$	1,593	1,305	689	1,008	392	985	707	1,572

The trend of the number of each population in the infectious disease model over time can be calculated, by using PYTHON programming to enter the parameters in Table 3 into the models. As illustrated in Figure 2, the number of infected and hospitalized patients grows over time and declines following adequate treatment, while the number of recovered patients increases throughout the whole stage. This result is consistent with the transmission mechanism of infectious diseases. From Figure 3, As medical and health care intervention gradually increases, δ of the corresponding model increases, the peak time of infection moved forward and the peak number of infected persons decreases accordingly. An adequate supply of medical resources is therefore critical to ending the outbreak quickly. In addition, early action on non-drug interventions are critical to epidemic control, and the lessons learned from epidemic control by WHO also show that early action will make significant progress in slowing and ultimately stopping outbreaks (39).

The model is programmed and the computation results are verified using the LINGO software and MATLAB YALMIP toolbox. When fairness is the main objective, the results of the ε-constraint method are shown in in Table 6: $X_{j,\min}^{t}=\;0.8$.

**Table 6:** Demand satisfaction rate of each epidemic area.

**Epidemic area**	**1**	**2**	**3**	**4**	**5**	**6**	**7**	**8**
Supply point	B	C	B	A	D	D	B	A
$X_{ij}^{t}$	1,400	1,000	591	900	225	875	509	1450
$X_{j}^{t}$	1	0.847	0.987	1	0.809	0.975	0.801	1

When the fairness and efficiency weights were 0.5, the results of the weighting sum method are shown in Table 7: $X_{j,\min}^{t}=\;0.8$.

**Table 7:** Demand satisfaction rate of each epidemic area.

**Epidemic area**	**1**	**2**	**3**	**4**	**5**	**6**	**7**	**8**
Supply point	A	A	D	C	A	B	B	B
$X_{ij}^{t}$	1,085	1,090	600	900	225	714	650	1,136
$X_{j}^{t}$	0.802	0.916	1	1	0.809	0.811	1	0.800

Generally speaking, the proposed dispatching and distribution model can satisfy the demand in each epidemic area to the greatest extent, meanwhile ensuring a fair allocation of materials among all the areas. Supplies will be delivered to each area from the nearest distribution center. Decision-makers can choose either objective based on their evaluations. The dual objective mixed-integer programming model, which takes into account both fairness and efficiency of the allocation of medical resources, also provides decision-makers flexibility to choose either objective based on their evaluations. Therefore, this work provides management insights for improving medical delivery decisions based on demand projections to rapidly respond to public health emergencies.

### 3.2. Experiments on the base case

#### 3.2.1. Effect of the lowest satisfaction rate

**Figure 4:**
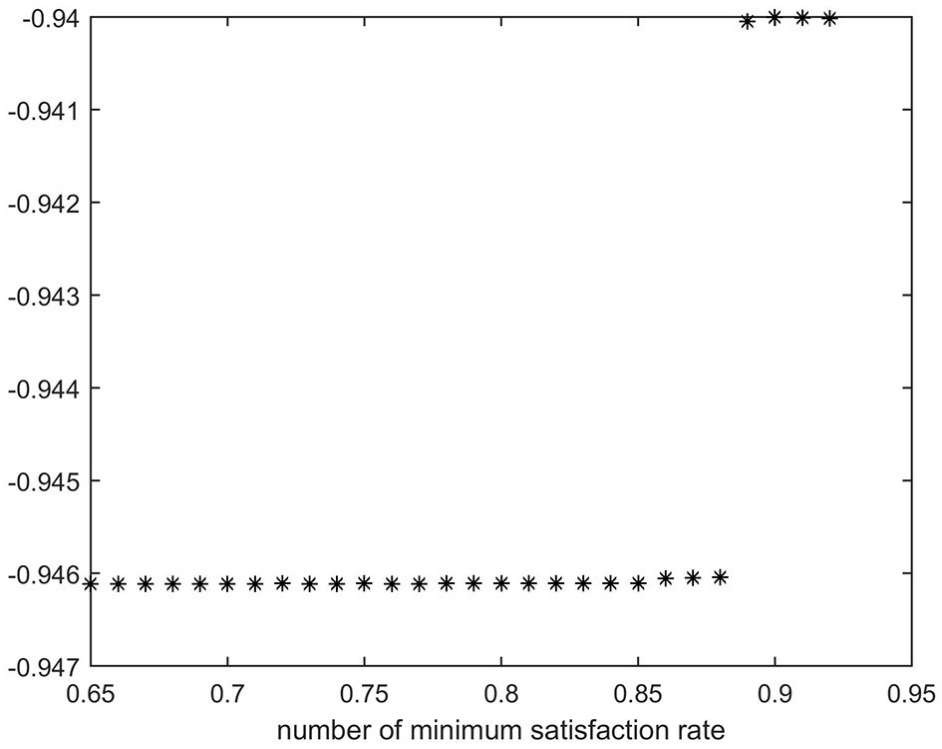
Impact of different minimum satisfaction rates on emergency rescue targets of the ε-constraint method.

This section primarily examines the effect of the difference in the minimum satisfaction rate of all epidemic areas on corresponding decisions, and the results of the ε-constraint method are presented in Table 8 and Figure 4.

**Table 8:** Impact of different minimum satisfaction rates on emergency rescue targets.

	**1**	**2**	**3**	**4**	**5**	**6**
$X_{j,\min}^{t}$	0.7	0.8	0.85	0.88	0.9	0.92
Total distance	4,045	4,045	4,045	4,815	4,815	3,285
Total satisfaction	0.94611	0.94611	0.94611	0.94605	0.94005	0.94001

The black dots in Figure 4 represent the value of objective function 1 at various levels of minimum satisfaction.

The results reveal that the value of the first objective function increases as the minimum satisfaction rate increases. Thus, the maximization of the weighted sum of the demand satisfaction rates shows a declining trend. This corresponds to the actual situation.

**Figure 5:**
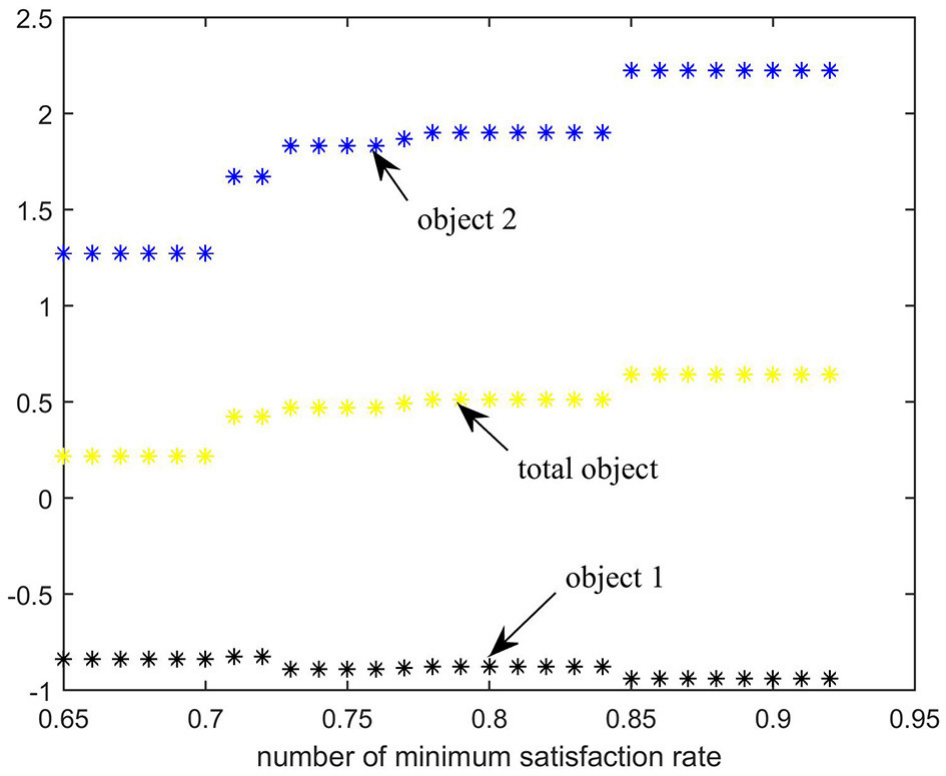
Impact of different minimum satisfaction rates on emergency rescue targets of the Weighting sum method.

When the fairness and efficiency weights are 0.5, the results of the weighting sum method are shown in Table 9 and Figure 5.

**Table 9:** Impact of different minimum satisfaction rates on emergency rescue targets.

	**1**	**2**	**3**	**4**	**5**	**6**
$X_{j,\min}^{t}$	0.7	0.8	0.85	0.88	0.9	0.92
Total distance	2,375	2,975	3,285	3,285	3,285	3,285
Total satisfaction	0.8370	0.8793	0.9401	0.9400	0.9400	0.9400

The black dots (object 1), blue dots (object 2), and yellow dots (total object) represent the values of objective 1, the normalized objective 2, and the weighted value of the total objective at various minimum satisfaction rates, respectively.

As shown in Figure 5, when the fairness and efficiency weights are set to 0.5, the total weighted satisfaction rate and vehicle travel distance in the epidemic area increase with the increment of the minimum satisfaction rate.

The value of the minimum demand satisfaction rate is extremely important, and decision-makers should assign appropriate values to parameters at various stages of the emergency process based on the actual situation, to ensure that the results obtained are feasible and effective.

#### 3.2.2. Effect of multiple supply points in an infected area

This section focuses on the influence of the different numbers of supply points on relevant decision-making in all epidemic areas where $X_{j,\min}^{t}=0.8$. When *K* (The upper limit of the number of supply points for transporting medical resources to epidemic areas) = 2, the satisfaction rate of each epidemic area is shown in Table 10.

**Table 10:** Satisfaction rate of each epidemic area and decision-making situation when *K* = 2.

**Epidemic area**	**1**	**2**	**3**	**4**	**5**	**6**	**7**	**8**
Supply point	A,B	A	B	D	B	B,D	B	C,D
$X_{ij}^{t}$	1,400	1,200	600	900	222	892	650	1,136
$X_{j}^{t}$	1	1	1	1	0.801	0.992	1	0.8

When *K* = 2, the weighted sum of the demand satisfaction rate is 0.9522, which is greater than when *K* = 1. When *K* = 2, the total travel distance is 4740 km, which is larger than the total travel distance of 4,045 km when *K* = 1. This trend is in line with common sense. Furthermore, when *K* = 3, the decision result is the same as when *K* = 2. Thus, even while the overall weighted satisfaction of residents in the epidemic area increases when supplies are delivered from two supply points, it happens that each supply point may deliver a small number of supplies, resulting in a waste of transportation resources. Therefore, for decision-makers, it is necessary to set up an appropriate number of supply points in the epidemic emergency medical logistics supply to effectively reduce rescue costs and improve the level of emergency services.

## 4. Conclusion

In comparison to traditional emergency logistics, emergency medical logistics (40) has three characteristics that raise the complexity and difficulty of solving logistical problems. To begin with, there is a dearth of demand-related information, such as the severity of the epidemic and the number of infected, as well as difficulties in marking distribution-related decisions. The incubation phase, in particular, results in a time delay in demand (41). Second, the disease can quickly spread from one location to another, resulting in a large-scale epidemic. Infection, recovery, and mortality rates typically vary across regions due to differences in individual physical conditions as well as habits, customs, and medical services provided by the hospitals in each region (42, 43). Third, unlike other forms of relief such as food, the substitutability of medical relief is imperfect. Specific medication cannot be completely replaced by another (44). This paper discusses how to allocate emergency supplies under two different infectious disease propagation scenarios. Specifically, this work contributes to the decision analysis of emergency medical logistics responses to public health emergencies in the following ways: First, based on the transmission rules of infectious diseases and local medical service capacity, we modify and develop a SEIHR (susceptible-exposed-infected-hospitalized-removed) model. These two models aim to forecast the time-varying demand for medical supplies in each epidemic area during the entire emergency rescue phase. Second, to find the optimal medical service allocation plan, we define emergency indicators for each epidemic area and propose a bi-objective optimization model to maximize the weighted demand satisfaction rate and minimize the total vehicle travel distance. Finally, we use linear weighting and the ε-constraint method to reformulate the bi-objective MILP model of emergency rescue logistics scheduling to a single-objective one. We also conduct numerical studies to examine the performance of the model.

### 4.1. Implications

Numerical results show that due to the hidden nature of asymptomatic infection, the number of infected people in infected areas will increase greatly. Decision-makers should assign appropriate values to parameters at various stages of the emergency process based on the actual situation, to ensure that the results obtained are feasible and effective. It is necessary to set up an appropriate number of supply points in the epidemic emergency medical logistics supply to effectively reduce rescue costs and improve the level of emergency services. Overall, this work provides managerial insights to improve decisions made on medical distribution as per demand forecasting for quick response to public health emergencies.

### 4.2. Limitations

Our study is hypothetical, without actual data. This study only evaluates how to predict the demand for medical aid resources and how to allocate resources based on the number of infected and hospitalized people in the epidemic areas. The supply points have also been fixed for the time being; however, these supply points can be adjusted continuously according to the number of supplies, location advantages, and the number of individuals serving in the epidemic area. Furthermore, the impact of population mobility and natural population growth rate on the spread of the epidemic is not considered in this work. Finally, the paper ignores the impact of material production capacity on decision-making.

In conclusion, the performance of emergency medical logistics may be improved significantly. Future research can take the material production supply chain, dynamic resource allocation based on locations of supplies, vehicle scheduling, and vehicle routing optimization problem into consideration. These factors help to establish a model that is more in line with the reality in medical resource allocation, thus more scientific and reasonable to solve practical problems.

## Data availability statement

The original contributions presented in the study are included in the article/supplementary material, further inquiries can be directed to the corresponding author.

## Author contributions

ZW: supervision. GJ: formal analysis, methodology, validation, and writing – original draft. XY, WC, and TY: visualization. QZ: writing – review and editing. BH: funding acquisition. All authors contributed to the article and approved the submitted version.
